# Unmanaged Diabetes and Elevated Blood Glucose Are Poor Prognostic Factors in the Severity and Recovery Time in Predominantly Hispanic Hospitalized COVID-19 Patients

**DOI:** 10.3389/fendo.2022.861385

**Published:** 2022-07-08

**Authors:** Sudip Bajpeyi, Ali Mossayebi, Helen Kreit, Sundar Cherukuri, Roshni A. Mandania, Jeannie B. Concha, Hyejin Jung, Amy Wagler, Akshay Gupte, Abhizith Deoker

**Affiliations:** ^1^ Metabolic, Nutrition and Exercise Research (MiNER) Laboratory, Department of Kinesiology, University of Texas at El Paso, El Paso, TX, United States; ^2^ Department of Internal Medicine, Texas Tech University Health Sciences Center, El Paso, TX, United States; ^3^ Paul L. Foster School of Medicine, Texas Tech University Health Sciences Center, El Paso, TX, United States; ^4^ Department of Public Health, University of Texas at El Paso, El Paso, TX, United States; ^5^ Department of Social Work, University of Texas at El Paso, El Paso, TX, United States; ^6^ Department of Mathematical Sciences, University of Texas at El Paso, El Paso, TX, United States; ^7^ Department of Neurosurgery, University Medical Center, El Paso, TX, United States

**Keywords:** COVID-19, hyperglycemia, type 2 diabetes (T2D), hispanic, unmanaged diabetes, blood glucose

Diabetes is considered to influence the severity and length of hospital stay (LOS) in COVID-19 patients. It is not known whether lack of diabetes management may magnify the severity and LOS in COVID-19 patients with diabetes. The purpose of the study was to determine the impact of unmanaged diabetes and acute glycemia on severity and LOS among hospitalized COVID-19 patients. This retrospective study used medical records from patients admitted to the University Medical Center, El Paso, TX with COVID-19. Glycemic control was assessed by fasting blood glucose (acute) and HbA1c (chronic); COVID-19 severity was measured by quick sepsis-related organ failure assessment (qSOFA) and the LOS was determined by the number of days spent in the hospital. Diabetes management with medication was self-reported by patients. There was no significant difference in severity and LOS between patients with and without diabetes. However, patients with unmanaged diabetes showed significantly greater severity and LOS compared to patients who managed diabetes. Patients with an elevated fasting blood glucose level also had a greater COVID-19 severity and LOS. In conclusion, unmanaged diabetes and blood glucose showed worsened severity and prolonged hospitalization in COVID-19 patients. Diabetes management should be considered in treatment of COVID-19 patients.

## Introduction

After the recognition of Coronavirus disease (COVID-19), the virus has rapidly spread and became a pandemic. Diabetes is a significant risk factor for elevated mortality rates in a wide array of diseases, including infectious diseases (1). Poor glycemic control and chronic hyperglycemia have been associated with morbidity and mortality (2). Diabetes and unmanaged glycemia were reported as significant predictors of severity and deaths in patients infected with viruses such as the 2009 pandemic influenza A virus (H1N1) and Middle East respiratory syndrome coronavirus (MERS-CoV) (3). While some studies reported no clear association between severity of COVID19 with diabetes (4, 5), others reported a greater risk for COVID-19 severity, length of hospital stay (LOS), and mortality among patients with diabetes (6–8). Patients with diabetes may be susceptible to more severe SARS-CoV-2 infection due to immune system dysfunction (9). Viral infections could also induce a diabetes state, or worsen hyperglycemia in people with diabetes, which may adversely influence prognosis (10).

Whereas an alarming proportion of the US population is diagnosed with diabetes, a significant proportion of the population is undiagnosed or does not manage diabetes. Yet, nearly 1 in 5 Americans with diabetes report that due to the increased financial constraints of the pandemic, they had to choose between buying food or buying medications and medical supplies required to manage their diabetes (11). Moreover, the Hispanic/Latino population is disproportionately impacted by diabetes, a population 50% more likely to have diabetes and 2.4 times more likely to die of COVID-19 than white Americans (11). Management of diabetes with hypoglycemic medication plays an important role in reducing diabetes-related diseases (12). Although it is clear from the present literature that both acute and chronic management of glycemia are important, it is not clear how unmanaged diabetes and acute glycemic control may affect the severity and the LOS in COVID-19 patients. Therefore, the purpose of this study was to determine the impact of unmanaged diabetes and acute glycemia on the severity and LOS among hospitalized, predominantly Hispanic and Latino, patients with COVID-19.

## Materials and Methods

### Study Design and Population

This is a single-center, retrospective, observational study that used data from the medical records of 858 confirmed COVID-19 cases admitted at the University Medical Center, El Paso, Texas between August, and September 2020. This study was approved by the institutional review board at the University of Texas at El Paso. From 858 patients who were initially included in this study, 369 patients met the inclusion criteria of having both hemoglobin A1c (HbA1c) and fasting blood glucose (FBG) data available at the time of admission (age 60.0 ± 16.6 years; BMI 30.3 ± 7.16 Kg/m^2^). Diabetes status was determined by following ADA guidelines for diabetes classifications of HbA1c level 6.5% or above at the time of the hospital admission. Patients with diagnosed diabetes (n=246) were further categorized into managed (n=182) and unmanaged (n=64) diabetes groups based on their self-reported diabetes management status with hypoglycemic medication (e.g. insulin sensitizer, insulin) at the time of hospitalization. In this study we have defined hyperglycemia in the context of diabetes following the American Diabetes Association criteria for diagnosis of diabetes using FBG≥126mg/dL or HbA1c≥6.5% (13). To evaluate the role of acute and chronic glycemic control on COVID-19 severity and LOS, all patients (n=364) were categorized into 4 groups based on their diabetes status evaluated by acute (FBG) and chronic (HbA1c) glycemic status at the time of hospitalization (**Figure 2**); Group 1 (G1): HbA1c<6.5%), FBG<126 mg/dl, Group 2 (G2): HbA1c<6.5%, FBG≥126 mg/dl, Group 3 (G3): HbA1c≥6.5%), FBG<126 mg/dl), and Group 4 (G4): HbA1c≥6.5%), FBG≥126 mg/dl).

### Data Collection

Clinical records and laboratory information for all patients hospitalized with confirmed COVID-19 were collected for this study. Information on patient demographics and health history data were collected. Acute and chronic glycemic control were assessed using FBG and HbA1c respectively during the time of hospital admission. The severity of the COVID-19 outcome was assessed by quick sepsis-related organ failure assessment (qSOFA) score (14) which identifies patients at high risk for in-hospital mortality based on systolic blood pressure, respiratory rate, and altered mental status from baseline. A qSOFA score of 2-3 indicates a high risk for in-hospital mortality compared to a lower score (15). The LOS was determined by the number of days spent in the hospital.

### Statistical Analysis

Data were analyzed using PRISM Graph Pad Software, version 8.0 (Graph Pad Software, La Jolla, California). Unpaired Student’s T-test and one-way ANOVA with Tukey *post hoc* analysis were used to compare means among two or more groups respectively, as appropriate. p-values less than 0.05 was considered statistically significant. All tables and figures represent data as mean and standard deviation (SD).

## Results

### No Difference in COVID-19 Severity and Length of Hospital Stay Based on Diabetes Status

The severity of COVID-19 measured by qSOFA score was not different between patients without and with diabetes groups (0.34 ± 0.49 vs. 0.28 ± 0.5; p=0.30) groups (**Table 1**). LOS was also similar between the two groups (8.15 ± 8.09 vs. 8.9 ± 9.53 days; p=0.47) (**Table 1**).

**Table 1:** Study subject characteristics.

	No Diabetes/Prediabetes	Diabetes	p value
**Number of subjects**	123	246	
**Sex (male/female)**	70/53	140/106	
**Age (years)**	59.41 ± 17.80	60.33 ± 14.51	0.60
**Ethnicity (Hispanic/Non-Hispanic)**	114/9	224/22	
**Body Mass Index (BMI) (kg/m^2^)**	27.23 ± 9.44	30.65 ± 6.22	0.18
**Fasting Blood Glucose (mg/dl)**	120.6 ± 42.01	214.9 ± 120.1	<0.0001^****^
**Glycated Hemoglobin (HbA1c) (%)**	5.9 ± 0.44	9.0 ± 2.40	<0.0001^****^
**Blood Pressure (Systolic) (mmHg)**	128.6 ± 25.89	135.3 ± 24.32	0.01*
**Blood Pressure (Diastolic) (mmHg)**	73.05 ± 14.32	75.8 ± 14.52	0.08
**Heart Rate (beats/min)**	93.86 ± 19.77	97.13 ± 17.65	0.11
**Hemoglobin (Hgb) (g/dL)**	13.05 ± 2.56	13.85 ± 6.84	0.21
**Alanine aminotransferase (ALT) IU/L**	48.55 ± 43.15	44.95 ± 48.71	0.49
**Aspartate aminotransferase (AST) IU/L**	58.2 ± 46.25	54.47 ± 57.30	0.53
**Blood Urea Nitrogen (BUN) (mg/dL)**	22.30 ± 18.25	23.17 ± 17.73	0.66
**Hospital stays (days)**	8.15 ± 8.09	8.88 ± 9.53	0.47
**qSOFA index**	0.34 ± 0.49	0.28 ± 0.50	0.30

qSOFA, quick sepsis-related organ failure assessment; Data Presented as mean ± SD. *p<0.05; ****p<0.0001.

### Lesser COVID-19 Severity and Shorter Duration of Hospital Stay In Patients Who Managed Diabetes With Hypoglycemic Medication

**Figure 1:**
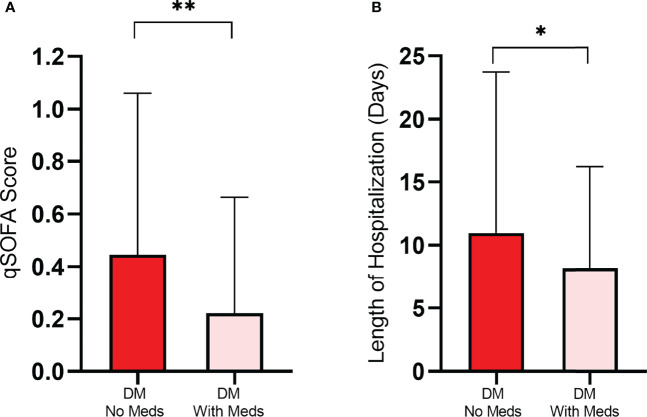
Severity **(A)** and length of hospital stay **(B)** among hospitalized COVID-19 patients with diabetes (DM) were significantly greater in patients with unmanaged diabetes (without medication) compared to patients who managed their diabetes with medication. Data presented as mean ± SD. *p<0.05, **p<0.01.

Patients who reported to have managed diabetes with hypoglycemic medication showed significantly lesser severity, assessed by qSOFA index, compared to those who reported to not manage diabetes (0.22 ± 0.44 vs. 0.44 ± 0.62; p<0.01) (**Figure 1A** and **Table 2**). Patients who managed diabetes also had significantly shorter LOS compared to patients with unmanaged diabetes (8.18 ± 8.04 vs. 10.98 ± 12.77 days; p<0.05) (**Figure 1B**). Consistent with these findings, when severity was compared among three groups including patients without diabetes (one-way ANOVA), unmanaged diabetes group showed significantly greater severity compared to patients with managed diabetes (0.22 ± 0.44 vs. 0.44 ± 0.61; p<0.05). Characteristics of the patients with managed and unmanaged diabetes is provided in **Table 2**.

**Table 2:** Study subject characteristics.

	Diabetes (managed)	Diabetes (not managed)	p value
**Number of subjects**	182	64	
**Sex (male/female)**	102/80	40/24	
**Age (years)**	61.88 ± 14.14	58.27 ± 15.39	0.09
**Ethnicity (Hispanic/Non-Hispanic)**	166/16	58/6	
**Body Mass Index (BMI) (kg/m^2^)**	31.04 ± 6.6	29.40 ± 4.75	0.07
**Fasting Blood Glucose (mg/dl)**	216.4 ± 101.5	160.9 ± 63.11	<0.0001^****^
**Glycated Hemoglobin (HbA1c) (%)**	9.4 ± 2.42	7.76 ± 1.85	<0.0001^****^
**Blood Pressure (Systolic) (mmHg)**	136.3 ± 24.32	132.4 ± 24.46	0.27
**Blood Pressure (Diastolic) (mmHg)**	76.08 ± 15.13	74.65 ± 12.56	0.50
**Heart Rate (beats/min)**	96.46 ± 18.54	98.63 ± 14.62	0.40
**Hemoglobin (Hgb) (g/dL)**	13.87 ± 7.83	13.74 ± 2.49	0.90
**Alanine aminotransferase (ALT) IU/L**	43.82 ± 49.70	47.79 ± 46.30	0.58
**Aspartate aminotransferase (AST) IU/L**	52.55 ± 56.49	59.22 ± 59.90	0.43
**Blood Urea Nitrogen (BUN) (mg/dL)**	23.23 ± 15.64	23.19 ± 22.93	0.99
**Hospital stays (days)**	8.18 ± 8.04	10.98 ± 12.77	0.04*
**qSOFA index**	0.22 ± 0.44	0.44 ± 0.61	0.002**

qSOFA, quick sepsis-related organ failure assessment; Data Presented as mean ± SD. *p<0.05; **p<0.01; ****p<0.0001.

### Role of Acute and Chronic Glycemic Control on COVID-19 Severity and Length of Hospital Stay

**Figure 2:**
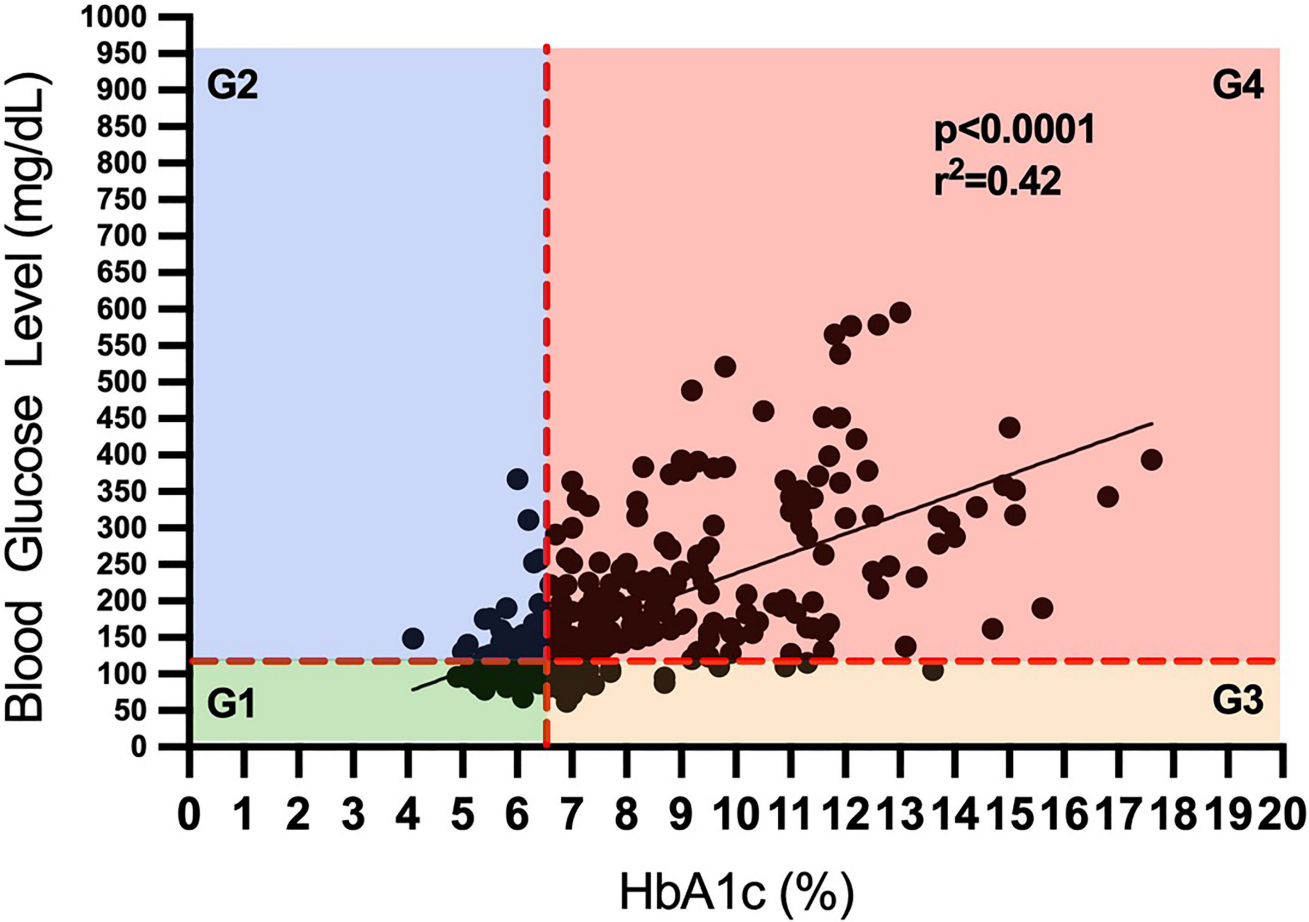
Categorization of patients for the diabetes status based on their fasting blood glucose and glycated hemoglobin level.

**Figure 3:**
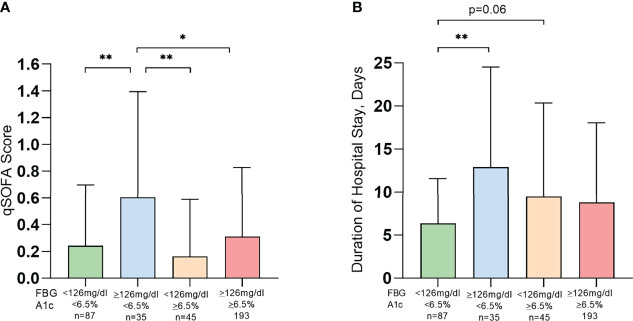
Patients with acute glycemia represented higher severity **(A)** and length of hospital stay **(B)** among hospitalized COVID-19 patients. FBG, fasting blood glucose; HbA1c, glycated hemoglobin; qSOFA, quick sepsis-related organ failure assessment; Data presented as mean ± SD. *p<0.05, **p<0.01.

Patients were categorized for the acute and chronic glycemic status (**Table 3** and **Figure 2**). Patients diagnosed with diabetes by FBG but not by HbA1c (G2) experienced a greater COVID-19 severity, measured by qSOFA, compared to the other 3 groups. (G2: 0.61 ± 0.79 vs. G1: 0.24 ± 0.46; p<0.01, G2: 0.61 ± 0.79 vs. G3: 0.16 ± 0.42; p<0.01, and G2: 0.61 ± 0.79 vs. G4: 0.31 ± 0.52; p<0.05) (**Figure 3A**). Patients in G2 also had significantly longer LOS compared to patients in G1 (G2: 12.91 ± 11.61 vs. G1: 6.36 ± 5.21 days; P<0.01) (**Figure 3B**).

**Table 3:** Characteristics of patients categorized into 4 groups based on their fasting blood glucose and glycated hemoglobin level.

Parameters	G1 (n = 87) FBG<126mg/dl HbA1c<6.5%	G2 (n = 35) FBG≥126mg/dl HbA1c<6.5%	G3 (n = 49) FBG<126mg/dl HbA1c≥6.5%	G4 (n = 193) FBG≥126mg/dl HbA1c≥6.5%
**Sex (male/female)**	48/39	21/14	28/21	104/89
**Age (years)**	56.47 ± 18.18^a^	66.29 ± 14.99^b^	60.43 ± 15.09	60.46 ± 14.29
**Ethnicity (Hispanic/** **Non-Hispanic)**	79/8	34/1	43/6	177/16
**BMI (kg/m^2^)**	29.87 ± 9.82	28.85 ± 5.37	30.51 ± 5.64	30.69 ± 6.38
**Medications (n (%))**	10 (11.5%)	6 (17%)	30 (61%)	137 (71%)
**FBG (mg/dl)**	103.7 ± 11.16^a^	165.9 ± 53.02^b^	105 ± 17.88^abc^	241.8 ± 110.2^c^
**HbA1c (%)**	5.8 ± 0.37^a^	5.9 ± 0.59^ab^	7.6 ± 1.91^c^	9.4 ± 2.40^d^
**Hospital stays (days)**	6.36 ± 5.21^a^	12.91 ± 11.61^b^	9.49 ± 10.86^ab^	8.81 ± 9.26^ab^
**qSOFA index**	0.24 ± 0.46^a^	0.61 ± 0.79^b^	0.16 ± 0.43^a^	0.31 ± 0.52^a^

BMI, body mass index; FBG, fasting blood glucose; HbA1c, glycated hemoglobin; qSOFA, quick sepsis-related organ failure assessment; Data presented as mean ± SD. ^a-d^ Values with different superscript letters in each row are significantly different (p<0.05).

## Discussion

This study aimed to gain insight into the impact of unmanaged diabetes and acute and chronic glycemic control on COVID-19 infection severity and recovery in COVID-19 patients. Our study reveals unmanaged diabetes and acute glycemia are key factors in COVID-19 severity and complications, among predominantly Hispanic and Latino populations.

We report no difference in COVID-19 severity and LOS among hospitalized patients based on diabetes status determined by HbA1c level in a predominantly Hispanic and Latino study population. Several meta-analyses have indicated greater severity of COVID-19 in patients with diabetes (16, 17) whereas, similar to our report, others have shown no difference in COVID-19 severity based on diabetes status (18) or established cardiovascular disease (19). Here using qSOFA, an objective clinical score identifying patients with suspected infection at greater risk for a poor outcome and increased in-hospital mortality, we report no difference in COVID-19 severity and LOS in a predominantly Hispanic/Latino population.

Although COVID-19 severity and LOS were not impacted by diabetes status in our study population, diabetes management status significantly impacted COVID-19 severity and LOS among COVID-19 patients with diabetes. Patients with unmanaged diabetes showed significantly greater severity of COVID-19 and longer LOS compared to those who managed diabetes with hypoglycemic medications. Our results are in line with other reports that indicated uncontrolled diabetes patients are at a greater risk for severe illness and COVID-19 severity (7, 20). Our data also clearly demonstrate the importance of diabetes management on COVID-19 severity and LOS. Although insulin therapy is an effective method for achieving glycemic targets and improving the clinical outcome of COVID-19 (20), a significant number of patients with diabetes report financial challenges to manage diabetes, which may have a greater impact on poor prognoses of patients with limited financial means (11).

Given that uncontrolled diabetes reflected worsened the severity of COVID-19, we further evaluated how acute and chronic glycemic control may impact COVID-19 severity and LOS irrespective of the diabetes status of the study population. Patients were categorized into 4 groups based on low or high acute (measured by FBG) and chronic (measured by HbA1c) glycemic control at the time of hospitalization. Patients with higher acute glycemia but controlled chronic glycemia (G2) had the greatest COVID-19 severity and longer LOS. Our results are consistent with recent studies that suggested uncontrolled glycemia to be a significant risk factor for the severity and morbidity of COVID-19 (21–23). Our results also suggested a lesser severity of COVID-19 with a high acute glycemia (FBG≥126mg/dL) in patients with relatively uncontrolled (HbA1c≥6.5%) chronic glycemia, compared to controlled (HbA1c ≤ 6.5%) chronic glycemia. This may perhaps be explained by better management of diabetes with hypoglycemic medication in the latter group (71% vs. 17%). In-hospital hyperglycemia has been known to be an important marker of poor clinical outcome, mortality, and longer stay in hospital in patients with or without a prior history of diabetes (21, 22, 24). It should be noted that the possible hyperglycemia with COVID-19 has also been suggested by underlying subclinical pulmonary remodeling (diabetic lungs) with COVID-19 infection that leads to hyperglycemia (25). Therefore, we cannot conclude whether a high blood glucose is a consequence of COVID-19. But it is clear from our data that unmanaged glycemic control is associated with worse COVID-19 severity and recovery time. Our study is the first study, to our knowledge, that investigated the effects of acute and chronic glycemic control concurrently on COVID19 severity and LOS. As glucose control helps prevent and control infections and their complications, well-managed blood glucose may lead to an improved outcome for patients with COVID-19.

Racial and ethnic disparities data indicate that COVID-19 has disproportionately infected Hispanic and Black communities. The Hispanic and Black populations are at a greater risk of experiencing COVID-19 severity, intensive care unit (ICU) admission, and risk of invasive mechanical ventilation (IMV) than non-Hispanic white patients (26). Diabetes is a prevalent disease among these racial/ethnic minority communities. Yet, many of them with diabetes often remain undiagnosed or are not able to properly manage their diabetes due to a lack of health insurance or financial resources to support their treatment. This circumstance is likely to expose them to the greater risk for COVID-19 severity and poor clinical outcomes. For example, El Paso has a predominately Hispanic and Latino population (82%) and is characterized as a low-income city where 69% of the population has a household income of less than $20,000 and 23% of the population has not graduated from high school (27). It is important to consider that our data represent a predominantly Hispanic and Latino population (88%), who are at a greater risk to develop diabetes (28). Our study findings highlight the importance of diabetes care in COVID-19 patients, particularly for the communities with a high prevalence of diabetes and limited resources.

One limitation of our study is that categorization of managed and unmanaged diabetes was done based on self-reported medication data. The information on previous diagnosis of diabetes and duration of diabetes were not available. Finally, we cannot completely rule out the impact of diabetes medication as we interpret our results on glycemic control impacting the severity and LOS. However, our data clearly demonstrate the impact of unmanaged hyperglycemia and acute glycemic control on the severity and length of recovery of COVID-19 in a predominantly Hispanic and Latino population.

In conclusion, our study clearly demonstrates that uncontrolled diabetes and acute glycemia worsened severity and the rate of recovery in COVID-19 patients. Our results highlight the importance of assessing, monitoring, and controlling blood glucose in hospitalized COVID-19 patients from the start, specifically for vulnerable populations already at risk of comorbidities. Therefore, during the pandemic of COVID-19, blood glucose management can facilitate the assessment of prognosis and early intervention of hyperglycemia to help improve the overall outcomes in the treatment of COVID-19.

## Data Availability Statement

This study used data from the medical records of confirmed COVID-19 cases. Any request for deidentified data should be directed to the corresponding author.

## Ethics Statement

This study was approved by the institutional review board at the University of Texas at El Paso.

## Author Contributions

SB conceived and designed the study; SB and AM wrote the first draft of the manuscript; all authors contributed to revision of the manuscript; HK, SC, and RM extracted the data, SB, AM, and AW analyzed the data, AW assisted with statistical analyses, all authors contributed to interpretation of the data. SB is responsible for the content of the article. Some of the results from this study were presented at the Texas chapter of American College of Sports Medicine (ACSM) conference, 2021 and American Diabetes Association (ADA) annual conference, 2021 and were published in abstract form. All authors contributed to the article and approved the submitted version.

## Conflict of Interest

The authors declare that the research was conducted in the absence of any commercial or financial relationships that could be construed as a potential conflict of interest.

## Publisher’s Note

All claims expressed in this article are solely those of the authors and do not necessarily represent those of their affiliated organizations, or those of the publisher, the editors and the reviewers. Any product that may be evaluated in this article, or claim that may be made by its manufacturer, is not guaranteed or endorsed by the publisher.
